# Knowledge and provision of misoprostol among pharmacy workers in Senegal: a cross sectional study

**DOI:** 10.1186/s12884-017-1394-5

**Published:** 2017-07-03

**Authors:** Kate Reiss, Katharine Footman, Eva Burke, Nafissatou Diop, Ramatoulaye Ndao, Babacar Mane, Maaike van Min, Thoai D. Ngo

**Affiliations:** 1 Health Systems Department, Marie Stopes International, 1 Conway Street, London, W1T 6LP UK; 2 Marie Stopes International Senegal, Sacre Coeur III, 10082 VDN Dakar, Senegal; 3 Population Council Senegal, Sacre Coeur Pyrotechnie, Appartement 2ème Etage à Droite, BP: 21027 Dakar, Ponty, Senegal

**Keywords:** Misoprostol, Post abortion care, Postpartum haemorrhage, Pharmacy, Maternal mortality, Senegal, Reproductive health

## Abstract

**Background:**

Making misoprostol widely available for management of postpartum haemorrhage (PPH) and post abortion care (PAC) is essential for reducing maternal mortality. Private pharmacies (thereafter called “pharmacies”) are integral in supplying medications to the general public in Senegal. In the case of misoprostol, pharmacies are also the main supplier to public providers and therefore have a key role in increasing its availability. This study seeks to understand knowledge and provision of misoprostol among pharmacy workers in Dakar, Senegal.

**Methods:**

A cross-sectional survey was conducted in Dakar, Senegal. 110 pharmacy workers were interviewed face-to-face to collect information on their knowledge and practice relating to the provision of misoprostol.

**Results:**

There are low levels of knowledge about misoprostol uses, registration status, treatment regimens and side effects among pharmacy workers, and corresponding low levels of training on its uses for reproductive health. Provision of misoprostol was low; of the 72% (*n* = 79) of pharmacy workers who had heard of the product, 35% (*n* = 27) reported selling it, though rarely for reproductive health indications. Almost half (49%, *n* = 25) of the respondents who did not sell misoprostol expressed willingness to do so. The main reasons pharmacy workers gave for not selling the product included stock outs (due to product unavailability from the supplier), perceived lack of demand and unwillingness to stock an abortifacient.

**Conclusions:**

Knowledge and availability of misoprostol in pharmacies in Senegal is low, posing potential challenges for delivery of post-abortion care and obstetric care. Training is required to address low levels of knowledge of misoprostol registration and uses among pharmacy workers. Barriers that prevent pharmacy workers from stocking misoprostol, including weaknesses in the supply chain and stigmatisation of the product must be addressed. Low reported sales for reproductive health indications also suggest limited prescribing of the product by health providers. Further research is needed to explore the reasons for this barrier to misoprostol availability.

## Background

Senegal has made progress toward reducing maternal mortality over the past two decades. However, with a maternal mortality ratio (MMR) estimated at 315 per 100,000 live births in 2015 [1], Senegal was not able to meet its Millennium Development Goal target of 190 by 2015 [2], and further advances are needed. While little data exists on the causes of maternal deaths in Senegal, post-partum haemorrhage is responsible for an estimated 34% of maternal deaths in sub-Saharan Africa [3]. Globally, a large proportion of deaths in early pregnancy are also due to unsafe abortion [4, 5]. It has been demonstrated that making misoprostol readily available in low-resource settings for management of postpartum haemorrhage (PPH) and post abortion care (PAC) can have a significant impact on reducing maternal deaths [6, 7]. Misoprostol is particularly appropriate for low resource settings as it does not require refrigeration and can be administered at the community-level by auxiliary midwives [8, 9].

Misoprostol was included in the World Health Organization’s Essential Medicines List (EML) in 2005 and was named for management of incomplete abortion and miscarriage in 2009 and for the prevention of PPH and induction of labour in 2011 [10–12]. In 2013, misoprostol was registered on the EML in Senegal as an anti-haemorrhagic and hemostatic product, and is available on the market for the treatment of duodenal and gastric ulcers, prevention of non-steroidal anti-inflammatory drugs (NSAID)-induced peptic ulcers, the prevention and treatment of PPH, PAC, intra-uterine foetal death (IUFD), and ripening of the cervix. The EML has yet to be widely disseminated in Senegal. Several brands of misoprostol exist on the market today, one of which, Misoclear®, is registered for gynaecological indications such as PPH and PAC. Recent research on misoprostol provision at the community level for PPH and PAC in Senegal has shown high levels of efficacy and acceptability [13, 14] and found it to be more cost effective for PPH prevention than oxytocin [15]. However, misoprostol remains underused [13, 14], and its availability in public and private health facilities is limited [16].

Misoprostol is also an abortifacient. Abortion is legal in Senegal only to save the mother’s life [17]. Evidence suggests that women recourse to unsafe procedures outside the health system when attempting to terminate pregnancies for other reasons [18], contributing to Senegal’s high rates of maternal morbidity and mortality [1, 19]. In some restrictive settings around the world, it has been documented that women are using misoprostol purchased over the counter as a safer alternative to high risk methods of pregnancy termination such as those undertaken by untrained providers in unhygienic settings [20, 21]. Awareness of misoprostol’s use for termination of pregnancy among providers and pharmacy workers is not documented in Senegal.

Global evidence indicates that private pharmacies (thereafter called “pharmacies”) and pharmacy workers are often the first and sometimes only point of contact with the health system and therefore play an important role in providing basic health information and services, including misoprostol, in many countries [22–27]. Pharmacies may be chosen because of their long opening hours, short waiting times, convenient locations, anonymity, and because they offer greater availability of medicines than public health facilities [26]. Despite this potential, there are concerns about the quality of care provided by pharmacies; pharmacy curricular may not include courses on the role of pharmacists as community health advisors and frontline staff may not be formally trained on medications, regimen and doses [26]. In Senegal, a University awarded diploma in pharmacy is a required to open and run a pharmacy, but not to sell medications from the outlet. Availability of misoprostol from pharmacies is also a concern, as pharmacists may be unwilling to stock and dispense a medication that can be used as an abortifacient [27].

Pharmacies are integral to the supply of medications in Senegal; selling misoprostol both to the general public and to providers. In 2014, the government opened a tender to purchase misoprostol for the public sector; however, at the time of writing, public providers were mostly accessing the product through pharmacies. To enable continued reductions in MMR in Senegal, it is important to ensure that misoprostol is widely available in pharmacies. This study seeks to understand knowledge and provision of misoprostol among pharmacies in Dakar, Senegal. Specifically, its objectives are (i) to document knowledge of misoprostol among pharmacy workers; (ii) to document the availability of and demand for misoprostol in the pharmacies; and (iii) to document self-reported provision practices by pharmacy workers.

## Methods

In September 2013, Marie Stopes International and the Population Council conducted a cross-sectional survey with pharmacy workers in Dakar, Senegal [18]. We obtained the list of all 557 registered pharmacies in the Dakar region (Dakar city and three suburbs located between 15 and 30 km from Dakar city centre) from the Order of Pharmacists, and used simple random sampling to select 110 pharmacies for inclusion in the study. Endorsement for the study was given by the Order of Pharmacists who contacted pharmacy workers in writing prior to data collection to endorse the study. Within each pharmacy, one worker was selected for a face-to-face interview. Selection criteria were (i) being involved in selling/distributing medicine; and (ii) being over 18 years of age. In each pharmacy, the owner or manager was approached first, and if they did not meet the eligibility criteria, for example if they did not directly sell or distribute medicine, the next most senior and eligible staff member was invited to participate. Pharmacy workers received an information sheet explaining the study, and were asked to give informed consent prior to interview. Interviewers were trained to explain the importance of the study and to ensure participants understood that their responses would be anonymised. Interviews took place in a setting within the pharmacy where the interview could not be overheard and where the interviewee felt comfortable providing information. A standardised, structured questionnaire was administered by a trained interviewer. The questionnaire collected information on pharmacy characteristics, staff background, knowledge and current practice relating to misoprostol as well as current frequency of misoprostol sales. Participants were able to refer to sales registers where needed. The questionnaire also included open-ended “Other” categories for some questions to collect answers not pre-coded, and respondents were given the opportunity to provide additional comments at the end of the interview. The instrument was pre-tested in eight pharmacies in Dakar.

Data were entered into an access database using Epi Info. Double data entry was used to check for data entry errors. Quantitative data were analysed descriptively using Stata 13 (StataCorp LP, College Station, TX, USA).

## Results

One hundred and ten structured interviews were completed, a response rate of 100%. Table 1 shows that about half (49%, *n* = 54) of respondents worked in pharmacies in Dakar city, and half (51%, *n* = 56) in pharmacies in the suburbs. The mean age of respondents was 41, and 63% (*n* = 69) of them were male. While overall, 82% (*n* = 89) had a relevant degree from a (public) university or private institution, data show heterogeneity in training by location: all respondents in Dakar city had some level of professional or university training whereas 36% (*n* = 20) of pharmacy workers in the suburbs had only received on-the-job training.

**Table 1:** Characteristics of respondents

	Dakar city		Suburbs^a^		Total	
	N	%	N	%	N	%
Total	54	49	56	51	110	100
Gender						
Male	31	57	38	68	69	63
Female	23	43	18	32	41	37
Age^b^						
22-30	11	21	7	13	18	17
31-40	12	23	28	53	40	38
41-50	18	34	14	26	32	30
51-65	12	23	4	8	16	15
Education						
Primary	0	0	1	2	1	1
Secondary	20	37	24	43	44	40
University degree	34	63	31	55	65	59
Size of pharmacy staff^c^						
2-3	13	25	23	42	36	33
4-6	32	60	27	49	59	55
7+	8	15	5	9	13	12
Position held						
Owner	24	44	22	39	46	42
Manager	5	9	10	18	15	14
Employee	25	46	23	41	48	44
Trainee	0	0	1	2	1	1
Training level^d^						
On the job training	0	0	20	36	20	18
Private institution	20	37	8	15	28	26
(Public) University degree	34	63	27	49	61	56

^a^Suburbs regroup pharmacies in the districts of Pikine, Guédiawaye and Rufisqu ^b^Data missing for one pharmacy worker in Dakar and three pharmacy workers in the Suburbs ^c^Data missing for one pharmacy worker in Dakar, one pharmacy worker in the Suburbs didn’t know the number of staff ^d^Data missing for one pharmacy worker in the Suburbs

### Knowledge and training of pharmacy workers

Table 2 shows that a high proportion of respondents had heard of misoprostol (72%, *n* = 79). Gastric and duodenal ulcer was the condition for which knowledge of registration and use of the product were highest. There was little awareness that misoprostol was registered for PPH prevention (1%, *n* = 1) and treatment (3%, *n* = 2), PAC (13%, *n* = 10), and other conditions. Knowledge of misoprostol’s reproductive health uses was highest for termination of pregnancy (38%, *n* = 30) and PAC (13%, *n* = 10), and lowest for conditions such as prevention (1%, *n* = 1) and treatment (4%, *n* = 3) of PPH, treatment of IUFD (1%, *n* = 1) and cervical ripening (3%, *n* = 2). Knowledge that misoprostol could be used for a condition did not translate into knowledge of the regimen. For example, 31% (*n* = 18) of the respondents who knew misoprostol could be used to treat gastric and duodenal ulcer reported that they did not know the regimen. Furthermore, 60% (*n* = 6) of those who knew misoprostol could be used for PAC reported not knowing its regimen and only 20% (*n* = 2) gave the correct total dose and route of administration (600mcg administered orally or 400mcg administered sublingually). Additionally, almost half of respondents that had heard of misoprostol could not name a side effect (Table 2).

**Table 2:** Pharmacy worker knowledge of misoprostol

	Number		Percent	
Respondent had heard of misoprostol	79		72	
*Knowledge of conditions*	*Used for*		*Registered for*	
	*N*	%	*N*	%
Duodenal or gastric ulcer	59	75	48	61
Prophylaxis of NSAID-induced peptic ulcers	5	6	4	5
Prevention of PPH	1	1	1	1
Treatment of PPH	3	4	2	3
PAC	10	13	10	13
Treatment for intrauterine fetal death (IUFD)	1	1	0	0
Cervical ripening	2	3	1	1
Termination of pregnancy	30	38	5	6
*Knowledge of storage instructions*	*N*		%	
Store away from heat	66		84	
Store away from moisture	22		28	
*Knowledge of side effects*				
Could not name any side effects	37		47	
Diarrhoea	6		8	
Nausea	4		5	
Fever/chills	3		4	
Weakness	5		6	
Bleeding	6		8	
Headaches	3		4	
Dizziness	11		14	
Abortion	10		13	

For misoprostol to be used effectively, it should be stored away from heat and moisture, but the majority of respondents were only aware of the need to keep misoprostol protected from heat (misoprostol in Senegal is packaged in moisture resistant blister packaging, however it is still recommended practice to minimise exposure to humidity).

While the majority of respondents that had heard of misoprostol reported ever receiving information or training on misoprostol, the predominant source of information was literature or brochures (45%, *n* = 22) (Table 3). Other sources of information on misoprostol included colleagues (20%, *n* = 10) and medical representatives or pharmaceutical companies (22%, *n* = 11). Just 10% (*n* = 5) of respondents indicated receiving information or training on misoprostol from a University. A low proportion of pharmacy workers had received training on post abortion care (12%, *n* = 6), while no respondents had received information about misoprostol use to prevent or treat PPH, cervical ripening or IUFD. Over two-thirds (67%, *n* = 33) had received information regarding the treatment of duodenal or gastric ulcer. 86% (*n* = 67) of respondents wanted more support or information about misoprostol, and 51% (*n* = 40) reported they do not feel confident providing misoprostol to end users. Among respondents desiring more information, the most commonly requested topics were the uses of misoprostol (37%, *n* = 25), its side effects (30%, *n* = 20) and dosage (28%, *n* = 19). However, 58% (*n* = 39) of respondents wanted training on all aspects of misoprostol.

**Table 3:** Training and training needs on misoprostol among pharmacy workers who had heard of the drug

	Number	Percent
Respondent had received information/training^a^	49	62
*Source of information/training*		
Colleague	10	20
Doctor	4	8
Medical representative/pharmaceutical company	11	22
Literature/Brochures	22	45
University	5	10
Client	2	4
Television or Internet	2	4
*Conditions trained in*		
Duodenal or gastric ulcer	33	67
Prophylaxis of NSAID-induced peptic ulcers	3	6
Prevention or treatment of PPH	0	0
PAC	6	12
Treatment IUFD	0	0
Cervical ripening	0	0
Respondent reported they do not feel confident providing misoprostol to end users^a, b^	40	51
Respondent wanted more information or support^a, b^	67	86
*Training topics needed*		
Uses of misoprostol	25	37
Storage of misoprostol	2	3
Dosage	19	28
Method of administration	5	7
Side effects	20	30
Complications	14	21
All aspects	39	58

^a^Among those respondents who had heard of misoprostol ^b^Data missing from one pharmacy worker

### Provision of misoprostol and reasons for not selling it

Table 4 shows that of the 79 respondents who had heard of misoprostol, 27 (35%) reported selling it. They sold misoprostol most commonly to men (56%, *n* = 15), married women (56%, *n* = 15) and unmarried women (41%, *n* = 11). No respondents reported selling misoprostol to adolescents. 30% (*n* = 8) of respondents reported selling misoprostol to health providers, most commonly to doctors (19%, *n* = 5) and midwives (7%, *n* = 3) (not shown). Sales were overwhelmingly reported to be for duodenal or gastric ulcer (63%, *n* = 17). Sale for reproductive health care uses was rarely mentioned.

**Table 4:** Self-reported provision of misoprostol among 27 pharmacy workers

	Number	Percent
Pharmacies sell misoprostol^a^	27	35
*Brands sold*	*Price per box [95%CI]* (West African CFA Franc)	
Cytotec (60 x 200mcg doses/box)	16,376	
	[13,824; 18,927]	
Misoclear (10 x 200mcg doses/box)	2162	
	[2149; 2176]	
Arthotec (30 x 200mcg doses/box)	5701	
	[5369; 6032]	
*Clients misoprostol sold to*	N	%
Health care providers	8	30
Married women	15	56
Unmarried women	11	41
Adolescents	0	0
Men	15	56
*Conditions misoprostol sold for*	N	%
Duodenal or gastric ulcer	17	63
Prophylaxis of NSAID-induced peptic ulcers	2	7
Prevention or treatment of PPH	0	0
Post abortion care	1	4
Treatment for intrauterine fetal death (IUFD)	0	0
Termination of pregnancy	1	4

^a^Among 79 respondents who had heard of misoprostol, data missing for one pharmacy worker

Just under half (49%, *n* = 25) of the 51 respondents who had heard of but didn’t sell misoprostol expressed interest in and willingness to stock the product. The most common reasons for not stocking misoprostol were lack of demand (56%, *n* = 30), unavailability of the product (46%, *n* = 24) and unwillingness to stock abortifacients (42%, *n* = 23).

### Demand for misoprostol

Whether they sold misoprostol or not, pharmacy workers reported having received requests for information about and/or purchase of the product. For example, 15% (*n* = 12) of study respondents who had heard of misoprostol reported receiving requests from health professionals for information about the product. The majority of the 12 (75%, *n* = 9) reported receiving requests from midwives. Providers most commonly wanted to know misoprostol’s indications (58%, *n* = 7) and its side effects (42%, *n* = 5).

Among the 27 pharmacy workers who reported selling misoprostol, 22% (*n* = 6) reported receiving requests to purchase misoprostol over the counter, i.e. without a prescription. No pharmacy worker reported selling it without prescription. Over the counter requests had been received for treatment of gastric ulcer (three respondents) and to terminate pregnancy (one respondent). Three respondents reported receiving clients who did not specify the indication.

Among the 52 respondents who said that they did not sell misoprostol, 21% (*n* = 11) reported experiencing a demand for the product from health professionals, and 71% (*n* = 37) reported demand from end users. A small number (8%, *n* = 3) said that the demand was for termination of pregnancy. However, the majority (62%, *n* = 23) reported not asking the indication for which clients wanted misoprostol.

## Discussion

This study found low levels of knowledge about misoprostol use, registration status, treatment regimens and side effects among pharmacy workers in Dakar, Senegal. Similar findings have recently been reported in Tanzania and Nigeria [28–30]. Although the majority of pharmacy workers had received some form of information or training on misoprostol, it was often only in the form of literature or brochures, and only on the treatment of duodenal or gastric ulcer. Pharmacy workers expressed high levels of desire for information or support on misoprostol.

As expected, given low levels of knowledge, the proportion of pharmacy workers stocking and providing misoprostol was also low, and very few respondents reported selling the product for reproductive health uses. However, half of the respondents who reported that they were not currently selling misoprostol said they were willing to stock it; their reasons for not selling the medicine were concerns about stock outs and perceived lack of demand. Recently, steps taken in Senegal to strengthen the supply chain have been successful in preventing stock outs of contraceptives [22]. Such efforts should also be applied to misoprostol.

Globally there is a critical shortage of highly-skilled healthcare workers such as physicians, nurses and midwives [31]. Expanding the role of pharmacists is recommended as one strategy for facilitating increased access to medications and reducing pressure on other areas of the health system [32, 33]. Pharmacists also have a vital role in assuring the effectiveness of medications and can prevent harm from incorrect use [32, 33]. As the scope and complexity of the pharmacist’s role increases, their competencies and skills need to be kept up-to-date though continued professional development activities [33]. In Senegal both public and private healthcare providers (midwives, nurses and physicians) procure medications from pharmacies. As well as being asked to sell misoprostol, 15% of pharmacy workers who had heard of the medication reported receiving requests from health professionals for information about it, further highlighting the importance of the pharmacist’s role and suggesting a lack of knowledge about misoprostol among other health professionals.

Oxytocin is the standard treatment for PPH, however lack of skilled health providers, lack of transportation and the medications’ need for refrigeration mean that it is often not accessible to women in Senegal, particularly in rural areas [13]. A recent trial in a rural area of Senegal found misoprostol administered by auxiliary midwives for PPH prevention both as effective as oxytocin and feasible to use [13, 15]. Similarly, misoprostol presents an opportunity for increased access to PAC in Senegal: a 2012 survey found that many poor and rural women don’t receive treatment for complications of abortion [18]. The survey found that some health posts (lowest level of nurse or midwife staffed public health facility) in rural areas of the country were not equipped to provide PAC but also noted that fear of criminal charges and stigma may inhibit access to existing services [18]. Unlike surgical PAC (manual or electric vacuum aspiration or dilation and curettage), misoprostol requires no surgical skills or equipment to administer making it feasible to scale up in resource scarce settings [34]. However, there is limited availability of misoprostol in facilities offering maternity services in Senegal [14] which may be due to lack of awareness of its reproductive health uses and limited supply of the drug.

Making misoprostol and information about its uses widely available in Senegal is important for increasing availability and quality of care for PAC and PPH. Pharmacies can increase awareness of and access to misoprostol and can support its correct use however the findings from this study demonstrate that these potential benefits were not being realised. Since this survey was conducted, in-service training and distribution of training materials for pharmacists and prescribers on misoprostol have been initiated in Senegal. Such interventions have the potential to address the knowledge gaps identified among these key groups. The rationale for increasing access to misoprostol must be highlighted to ensure that pharmacy workers recognise the contribution they can have to reducing maternal mortality in the country.

One of the reasons pharmacy workers gave for not stocking misoprostol was that they were unwilling to stock an abortifacient. This may be due to objection to abortion as reported in other countries [35]. In Senegal, in cases of conscientious objection, pharmacists are required to refer the client to another pharmacist or healthcare provider. Other reasons for this unwillingness to stock misoprostol could be lack of knowledge of the product’s registration status and of its legal indications. Wide dissemination of the EML could help de-stigmatise the product and fully inform providers and pharmacist workers of its life-saving properties for women experiencing PPH or in need of PAC [6]. In addition to these actions, further research is required to better understand the knowledge, attitudes and practice of health providers towards misoprostol and to identify barriers that could explain the low levels of prescription of the product.

A strength of this study is the unusually high participation rate despite the lack of financial incentive. It is likely that the endorsement from the Order of Pharmacists, the anonymous nature of the interviews and the explanation by interviewers about the importance of the study played a role in motivating pharmacy workers to take part. The study has a number of limitations. 1) The study was limited to Dakar where transport links are good, and additional research will be needed to assess misoprostol knowledge and provision in other regions of Senegal. 2) It is not clear whether the practice of respondents relate only to individual practice or to a pharmacy-wide policy. 3) Reporting on misoprostol sales and demand for misoprostol medications and information may have been subject to recall bias. Respondents were asked to report on their current practices however misoprostol may be a slow-moving product which could make recall more difficult. 4) Some of the findings may have been subject to reporting bias i.e. respondents may have given answers that they believe are politically correct and/or legally safe. More than one third of pharmacists reported knowing that misoprostol could be used for abortion and may therefore have been hesitant to report stocking or selling it. Underreporting of abortion practices in restrictive countries is known to be high due to fear of prosecution or stigma [36–38]. In this survey, a number of behaviours may be underreported including off-label provision, provision to adolescents, provision for termination of pregnancy (abortion is legal in very few circumstances and pharmacists are not legally allowed to provide them) and provision for PAC (which many perceive to be illegal). It is also possible that provision to married clients and for gastric ulcer are over reported. Further exploration is needed into the possibility that awareness of the link between misoprostol and abortion is inhibiting pharmacy worker’s willingness to stock and sell the product. Research methodologies such as mystery clients, which minimize pharmacy workers’ self-reporting bias, could be used to explore provision practices further. In-depth interviews could also be used to gain deeper understanding of pharmacy workers’ views about the use of misoprostol as an abortifacient and whether stigma affects provision practices.

## Conclusions

There is an urgent need to boost access to misoprostol in Senegal as a strategy for increasing availability and quality of care for PAC and PPH. Educating pharmacists and pharmacy workers about misoprostol, its indications for use and the correct dosages for each indication, could help efforts to improve access to this important reproductive health commodity in Senegal. Pharmacies are a key component of the supply chain of medications and their role in the scale-up of misoprostol must be clarified promptly.

## Abbreviations

EML: Essential Medicines List
IUFD: Intrauterine Foetal Death
MMR: Maternal Mortality Ratio
NSAID: Non-steroidal anti-inflammatory drugs
PAC: Post Abortion Care
PPH: Postpartum Haemorrhage
